# Reporter genes confer new-to-nature ornamental traits in plants

**DOI:** 10.1093/hr/uhac077

**Published:** 2022-04-11

**Authors:** Guoliang Yuan, Haiwei Lu, David J Weston, Sara Jawdy, Timothy J Tschaplinski, Gerald A Tuskan, Xiaohan Yang

**Affiliations:** 1 Biosciences Division, Oak Ridge National Laboratory, Oak Ridge TN 37831, USA; 2 The Center for Bioenergy Innovation, Oak Ridge National Laboratory, Oak Ridge, TN 37831, USA

## Abstract

This manuscript has been authored by UT-Battelle, LLC under Contract No. DE-AC05-00OR22725 with the U.S. Department of Energy. The United States Government retains and the publisher, by accepting the article for publication, acknowledges that the United States Government retains a non-exclusive, paid-up, irrevocable, worldwide license to publish or reproduce the published form of this manuscript, or allow others to do so, for United States Government purposes. The Department of Energy will provide public access to these results of federally sponsored research in accordance with the DOE Public Access Plan (http://energy.gov/downloads/doe-public-access-plan).

##  

Dear Editor,

Ornamental plants (trees, shrubs, and herbs) beautify our urban and rural environments, enrich the quality of human life, and represent a vital component of the horticultural industry. The introduction of novel plant varieties and cultivars is critical to the ornamental horticultural industry [1]. To develop ornamental plants with desirable traits, different approaches, such as ploidy manipulation, interspecific hybridization, and physical/chemical mutagenesis, have been used for decades [2, 3]. However, the breeding of new ornamental varieties of trees and shrubs is a time- and labor-consuming process, because these plants may have a long juvenile growth period, large physical size, or altered floral structures, and consequently require long-term observations, large areas for progeny testing, and/or special equipment for pollination and/or seed collection [4].

The utilization of genetic transformation to transfer genes cannot only expand the available toolkits for plant breeders, but also save considerable time in the breeding process [5]. For instance, Chin et al., generated brilliant green fluorescent *Petunia* plants by using a potent fluorescent protein eYGFPuv [6]. Furthermore, we recently successfully demonstrated the use of this eYGFPuv into additional herbaceous and woody plants, including tobacco (*Nicotiana benthamiana* and *Nicotiana tabacum*), *Arabidopsis thaliana*, poplar clone “717” (*Populus tremula* × *alba* INRA “717-1B4”), and citrus rootstock (*Citrus sinensis* “Washington” sweet orange × *Poncirus trifoliata*; *Carrizo citrange*) [7]. Similarly, the heterologous engineering of betalain pigments in plants have been achieved in a variety of plants. For example, the heterologous production of betalain pigments was reported in food crops tomato (*Solanum lycopersicum*), potato (*Solanum tuberosum*), eggplant (*Solanum melongena*), and ornamental petunia (*Petunia* sp.) by coexpressing three genes of the elucidated betalain biosynthetic pathway [8]. Along these same lines, a new reporter called RUBY was recently created. This reporter converts tyrosine to vividly red betalain, and was used as an effective selection marker for transformation events in both rice (*Oryza sativa*) and *A. thaliana* [9]. Considering that plant color is one of the most important ornamental traits, heterologous betalain production may offer exciting opportunities for creating new horticultural value for consumers, producers, and suppliers of ornamental plants [8]. However, the heterologous engineering of betalain pigments remains to be elucidated in woody plants. We, therefore, applied the reporter RUBY in the woody plant poplar in this study.

Using gblocks from Integrated DNA Technologies (IDT), we created the RUBY vector pAXY0003 containing 35S:RUBY using Gibson cloning [9]. After *Agrobacterium*-mediated tobacco leaf infiltration, we observed strong red pigment in the leaf of *N. benthamiana*, indicating the 35S:RUBY worked efficiently in transient expression. Since we already demonstrated the efficacy of eYGFPuv in tobacco leaves, we then combined the features of both eYGFPuv and RUBY reporters in order to enable color change under different lighting conditions. As such, we created the first dual reporter system pAXY0004 containing 35S:RUBY and 35S:eYGFPuv. Through tobacco leaf infiltration, we were able to observe brilliant green fluorescence and red pigment simultaneously in the infiltration area, suggesting that this dual reporter system worked efficiently in transient expression.

**Figure 1:**
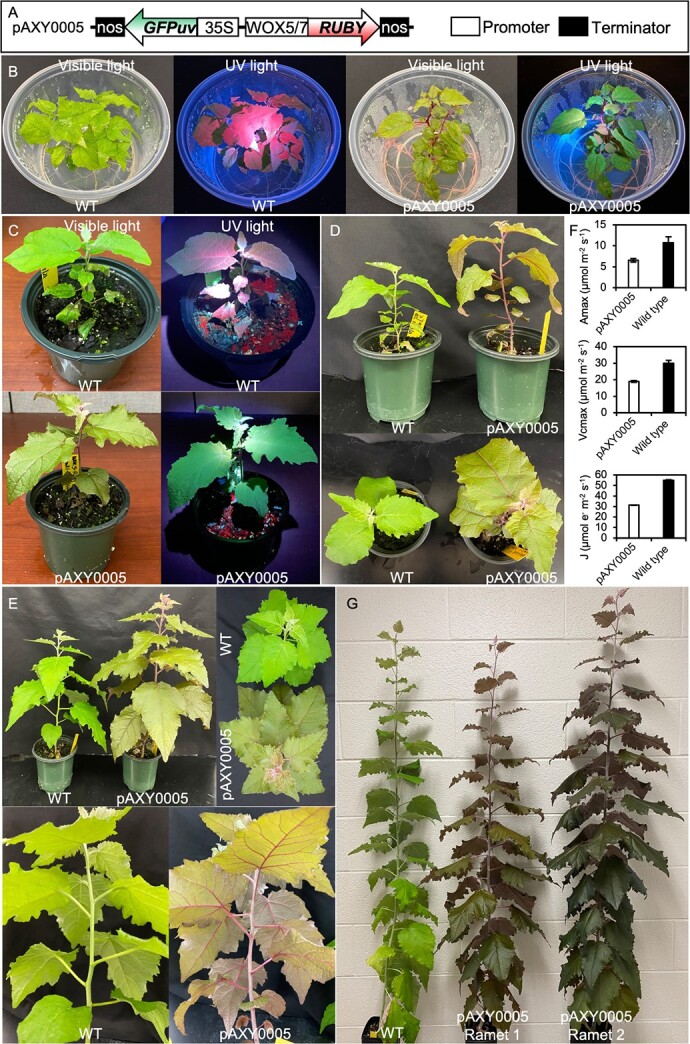
Visualization of eYGFPuv and RUBY at the whole plant level of poplar. (A) Schematic of the eYGFPuv and RUBY overexpression vector, pAXY0005. (B) Visualization of eYGFPuv and RUBY in selection medium of poplar transformation. (C) Visualization of eYGFPuv and RUBY in three-week-old, soil grown poplar, cultured in greenhouse. (D) Visualization of RUBY in four-week-old, soil grown poplar, cultured in greenhouse. (E) Visualization of RUBY in seven-week-old, soil grown poplar, cultured in greenhouse. (F) Comparison of photosynthesis between wild type poplar and transgenic line pAXY0005 (ten-week-old plants). Bars represent the mean values for maximal rates of net photosynthesis (Amax) taken at saturating light and CO_2_ levels, the maximal rate of electron transport (Jmax) and the maximal rate of Rubisco carboxylation (Vcmax). Error bars are mean values ± SD calculated from n = 3. (G) Visualization of RUBY in 16-week-old, soil grown poplar, cultured in greenhouse.

We then examined the efficacy of 35S:RUBY in a woody plant through the use of stable transformation in poplar clone “717” [7]. We were able to see red callus in the RUBY transformation. However, we were unable to recover any shoots from these red calluses in shoot induction medium. One possibility is that the high expression level of 35S:RUBY inhibited the shoot induction during RUBY transformation. In parallel, we created another dual reporter construct pAXY0005 in which we used a constitutive 35S promoter to drive the expression of eYGFPuv and the promoter of Potri.008G065400 in *Populus trichocarpa* to drive the expression of *RUBY* (Fig. 1A). The *Populus* gene Potri.008G065400 is homologous to *Arabidopsis* genes *WOX5* (AT3G11260) and *WOX7* (AT5G05770), both of which have been shown to be specifically expressed in regeneration-competent cells during *de novo* root formation in *Arabidopsis* [10]. We chose the promoter of Potri.008G065400 to create a tissue-specific construct that allows for *RUBY* expression in regeneration-competent cells during early organogenesis in poplar. After *Agrobacterium*-mediated stable transformation in poplar clone “717”, multiple independent transgenic events exhibiting green fluorescence were successfully selected under UV light in rooting medium (Fig. 1B). Interestingly, in one of these transgenic events, the vivid red pigment was observed at the whole-plant level, especially in root, stem, petiole, bark, and leaf veins (Fig. 1B). After multiple rounds of propagation in rooting medium, we finally produced more than 26 ramets from this initial event. We were able to continually observe homogeneous and uniform traits of RUBY and eYGFPuv in all ramets, indicating the stability of these novel ornamental traits during propagation. Next, we transferred multiple ramets into soil for greenhouse observation. After three weeks in soil, we observed typical phenotypes of RUBY and eYGFPuv under visible light and UV light, respectively, indicating that the traits of RUBY and eYGFPuv are robust and stable (Fig. 1C). As the plants grow, the traits of RUBY became more apparent in the larger leaves and longer stems due to the accumulation of red pigment (Fig. 1D and E). Analysis of photosynthesis in leaves from three ramets of transgenic line pAXY0005 showed that the maximal rates of net photosynthesis (Amax), electron transport (Jmax) and Rubisco carboxylation (Vcmax) were lower in the transgenic plants than the wild type plants (Fig. 1F). There is no noticeable difference in the growth rate of wild type and transgenic plants by week 16 (Fig. 1G). Notably, we observed both phenotypes of RUBY and eYGFPuv in multiple ramets cultured in greenhouse. These results support that the traits of RUBY and eYGFPuv can be easily maintained and stably spread in both tissue culture and greenhouse conditions.

In summary, we created an ornamental poplar genotype harboring novel decorative traits using reporter-gene-based transformation for the first time in woody plants. The pattern of RUBY phenotype of the transgenic line pAXY0005 could be resulted from the specificity of the promoter of Potri.008G065400 or of the insertion of T-DNA to a special location of the poplar genome, or both. It is possibly, though, the location of the insertion played a main or a larger role, as only one of the four recovered pAXY0005 events with strong eYGFPuv expression showed the RUBY phenotype (Data not shown). Nonetheless, our results demonstrated the potential of using RUBY and eYGFPuv to create novel decorative traits. Besides, the lower photosynthesis rate of transgenic line pAXY0005 could be a desired feature for indoor ornamental plants. Given that RUBY and eYGFPuv have been successfully applied in various plant species, we propose that this dual reporter system can be directly applied in other herbaceous and woody plant species in order to develop novel ornamental plants. In comparison with the conventional ornamental breeding tools, firstly, this approach dramatically decreased the cultivar creation period with easily selectable reporters. Secondly, the ornamental traits are predictable, designable and manipulatable. Technically, the desired traits can be either expressed at the whole-plant level using a constitutive promoter (like 35S or ubiquitin promoter) or in the specific plant tissue or organ (e.g. flower, leaf, and root) using a tissue-specific promoter, which is usually unachievable using conventional ornamental breeding techniques. Thirdly, the application of this approach is flexible and adjustable. In addition to RUBY and eYGFPuv, many other colorful reporters, such as chromoproteins, can be potentially incorporated into this system. For example, many eukaryotic chromoproteins, such as, mRPF1, amilCP and amilGFP have been used for bacterial synthetic biology, because these chromoproteins can be directly detected by the naked eye without requiring pretreatment [11]. Notably, the application of these chromoproteins in plants remains to be studied. Hence, with the exploration of new colorful reporters, the desired ornamental traits can be expanded through gene stacking. Finally, these colorful trees might also be a good vehicle to gain public acceptance of genetically modified woody plants.

## Data Availability

The plasmids will be available at Addgene (http://www.addgene.org). The construct data were deposited in Dryad (doi:10.5061/dryad.gf1vhhmrr).

## References

[1] Seaton K , Bettin A, Grüneberg H. Horticulture: Plants for People and Places. In: Dixon GR, Aldous DE, eds. Volume 1: Production Horticulture. Springer: Netherlands, 2014,435–63.

[2] Alix K , Gérard PR, Schwarzacher T et al. Polyploidy and interspecific hybridization: partners for adaptation, speciation and evolution in plants. Ann Bot. 2017;120:183–94. 28854567 10.1093/aob/mcx079 PMC5737848

[3] Ibrahim R , Ahmad Z, Salleh S et al. Mutation Breeding in Ornamentals. In: Ornamental Crops. Handbook of Plant Breeding; Van Huylenbroeck, J., Ed.; Springer: Dordrecht, The Netherlands, 2018, pp. 175–211. https://link.springer.com/chapter/10.1007/978-3-319-90698-0_8.

[4] Lebedev VG , Lebedeva TN, Chernodubov AI et al. Genomic selection for Forest tree improvement: methods. Achievements and Perspectives *Forests*. 2020;11:1190.

[5] Giovannini A , Laura M, Nesi B et al. Genes and genome editing tools for breeding desirable phenotypes in ornamentals. Plant Cell Rep. 2021;40:461–78. 33388891 10.1007/s00299-020-02632-x PMC7778708

[6] Chin DP , Shiratori I, Shimizu A et al. Generation of brilliant green fluorescent petunia plants by using a new and potent fluorescent protein transgene. Sci Rep. 2018;8:16556. 30410086 10.1038/s41598-018-34837-2 PMC6224394

[7] Yuan G , Lu H, Tang D et al. Expanding the application of a UV-visible reporter for transient gene expression and stable transformation in plants. Horticulture Research. 2021;8:234. 34719678 10.1038/s41438-021-00663-3 PMC8558336

[8] Polturak G , Grossman N, Vela-Corcia D et al. Engineered gray mold resistance, antioxidant capacity, and pigmentation in betalain-producing crops and ornamentals. Proc Natl Acad Sci. 2017;114:9062. 28760998 10.1073/pnas.1707176114 PMC5576821

[9] He Y , Zhang T, Sun H et al. A reporter for noninvasively monitoring gene expression and plant transformation. Horticulture Research. 2020;7:152. 33024566 10.1038/s41438-020-00390-1 PMC7502077

[10] Sugimoto K , Temman H, Kadokura S et al. To regenerate or not to regenerate: factors that drive plant regeneration. Curr Opin Plant Biol. 2019;47:138–50. 30703741 10.1016/j.pbi.2018.12.002

[11] Liljeruhm J , Funk SL, Tietscher S et al. Engineering a palette of eukaryotic chromoproteins for bacterial synthetic biology. J Biol Eng. 2018;12:8. 29760772 10.1186/s13036-018-0100-0 PMC5946454

